# Erythema nodosum as the initial presentation of leprosy in a 15-year-old boy: a case report on Ethiopia’s persistent elimination challenges

**DOI:** 10.3389/fmed.2024.1490279

**Published:** 2025-01-23

**Authors:** Kedir Negesso Tukeni, Tadele Molla Aga, Elias Ababulgu Abadiko, Idosa Taso Mamo, Tamirat Godebo Woyimo, Eyob Tarekegn Biru, Abdo Kedir Abafogi, Esayas Kebede Gudina

**Affiliations:** Jimma University, Jimma, Ethiopia

**Keywords:** leprosy, multibacillary leprosy, lepra reaction, erythema nodosum leprosum, endemic disease, Ethiopia

## Abstract

Leprosy remains a significant public health concern in Ethiopia, with challenges in timely diagnosis and management contributing to disabilities. This case report details a 15-year-old boy who presented with diffuse nodular and bullous skin lesions, peripheral neuropathy, and systemic symptoms, ultimately diagnosed with multibacillary leprosy confirmed by a positive skin smear for acid-fast bacilli. A 15-year-old immunocompetent boy presented to the emergency department with a 2-week history of diffuse, non-pruritic skin lesions, joint swelling, numbness, and tingling sensation. The patient was febrile on arrival. Physical examination revealed an erythematous nodular skin lesion over the face and hand bilaterally and a non-blanching bullous lesion over the right leg. The left hand was swollen and tender to touch, with a superficial collection, suggestive of an abscess. There was also a hypopigmented skin lesion over the medial aspect of the left leg and thigh bilaterally with loss of sensation. There were bilateral ulnar and radial nerve enlargement, saddle nose, and tenderness at the olecranon area. The skin smear was positive for acid-fast bacilli. The patient was initially treated with corticosteroids and antibiotics, followed by referral for multidrug therapy, and was advised for further follow-up for treatment response. The case describes the critical role of early recognition and multidisciplinary management in leprosy, particularly in endemic areas. The complexity of the presentation demonstrates the need for ongoing vigilance among healthcare providers. Public health initiatives aimed at improving diagnostic capabilities and expanding leprosy services are vital for reducing the disease burden in countries such as Ethiopia.

## Introduction

Leprosy is a chronic infectious disease caused by *Mycobacterium leprae* (1). Recent literature indicates that leprosy is caused by both *Mycobacterium leprae* and *M. lepromatosis*, with *M. leprae* being the primary etiological agent of the disease. It often affects the skin and peripheral nerves, resulting in a variety of skin lesions and nerve damage that can lead to impairments, deformities, and lepra reactions, characterized by cutaneous and systemic involvement, caused by the sudden alteration in immune response. Up to 50% of leprosy patients experience one of the three types of lepra reactions: type 1, type 2, or the Lucio phenomenon. Type 2 lepra reaction, also known as erythema nodosum leprosum or ENL, is a recurring multisystem illness brought on by immune complex deposition. Lepromatous leprosy (LL) or borderline leprosy (BL) patients are the main victims of ENL, and women’s hormonal changes during puberty, pregnancy, or lactation are the risk factors for the development of this reaction. It begins as abrupt, sensitive, erythematous nodules on the face or limbs, which can occasionally develop into bullous or necrotic forms. These nodules last 1–2 weeks; however, they may persist for months. To avoid serious neurological consequences and impairment, early diagnosis and treatment are essential (2–4).

The introduction and widespread use of multi-drug therapy (MDT) since the 1980s have greatly reduced the global burden of the disease. For instance, the number of leprosy cases worldwide dropped from 5.2 million in 1985 to 180,618 by the end of 2013; however, the actual number of newly diagnosed cases in 2013 was much higher at 215,656 cases (2).

Despite these advancements, leprosy continues to be a public health challenge. In 2017, 210,671 new cases were reported from 150 countries, with a detection rate of 2.77/100,000 population and a recorded prevalence rate of 0.25/10,000 population (3). India, Brazil, and Indonesia accounted for 80.2% of new cases globally, while in Africa, the highest number of new cases was reported from the Democratic Republic of the Congo, Ethiopia, and Nigeria (3). Leprosy remains a serious public health problem in Ethiopia due to its associated morbidity and socioeconomic impacts (4). The new case detection rate (NCDR) in Ethiopia for the last 10 years has been between 2,400 and 4,000, considered highly endemic. With this many new cases each year, Ethiopia is still considered one of the 23 global priority countries from 2014 to 2023 (5). To address these challenges, Ethiopia established the National Leprosy Control Program in 1956, with the goal of improving access to leprosy services, enhancing early detection and treatment, reducing stigma against people suffering from the disease, and ensuring the sustainability of leprosy control initiatives (6). As part of this effort, a leprosy mapping initiative was launched to identify higher-burden districts and boost leprosy control efforts. In 2013, the country recorded 4,374 leprosy cases, with 8.3% of patients presenting with disability grade II at diagnosis and 10.6% being children under 15 years of age, indicating ongoing community transmission (2).

Anecdotal reports from leprosy supervisors and clinical workers have revealed a significant gap in healthcare personnel’s knowledge and skills regarding leprosy case management. A study found that 82% of healthcare providers had limited knowledge of leprosy, with only 18% being able to correctly diagnose leprosy (7). This knowledge gap could lead to delayed diagnosis and mismanagement, increasing the risk of disabilities. Identifying leprosy hotspots through epidemiological mapping remains crucial to improving early diagnosis and treatment. Enhancing training for healthcare providers and prioritization of resources in higher-burden areas are essential to better manage leprosy in Ethiopia (5).

This case report describes a 15-year-old immunocompetent boy who presented with diffuse, nodular, and bullous skin lesions, peripheral nerve involvement, and systemic symptoms, ultimately diagnosed as multibacillary leprosy confirmed by a positive skin smear for acid-fast bacilli. The presentation, which was complicated by a type 2 lepra reaction (erythema nodosum leprosum), involved erythematous nodules, hypopigmented lesions with sensory loss, and nerve enlargement. Initial treatment included corticosteroids and antibiotics, followed by referral for multidrug therapy. The case underscores the need for improved healthcare provider training for early diagnosis and comprehensive care to reduce the burden of leprosy in endemic areas such as Ethiopia.

## Case description

A 15-year-old immunocompetent boy presented to the emergency room with a 2-week history of diffuse, non-pruritic skin lesions, wrist joint swelling, chills, and sensory disturbances including numbness and tingling sensation. Before his current complaints, he had a history of sensory disturbance, numbness, and tingling sensation for a year duration though the patient did not visit any local healthcare facilities. Upon his arrival at our emergency room, the patient was febrile and reported severe pain in his extremities. He denied any gastrointestinal symptoms such as diarrhea or abdominal pain, as well as respiratory symptoms such as cough, history of shortness of breath, chest pain, night sweating, or weight loss. There was no history of allergy, recent sore throat, or similar illness in the household.

**Figure 1:**
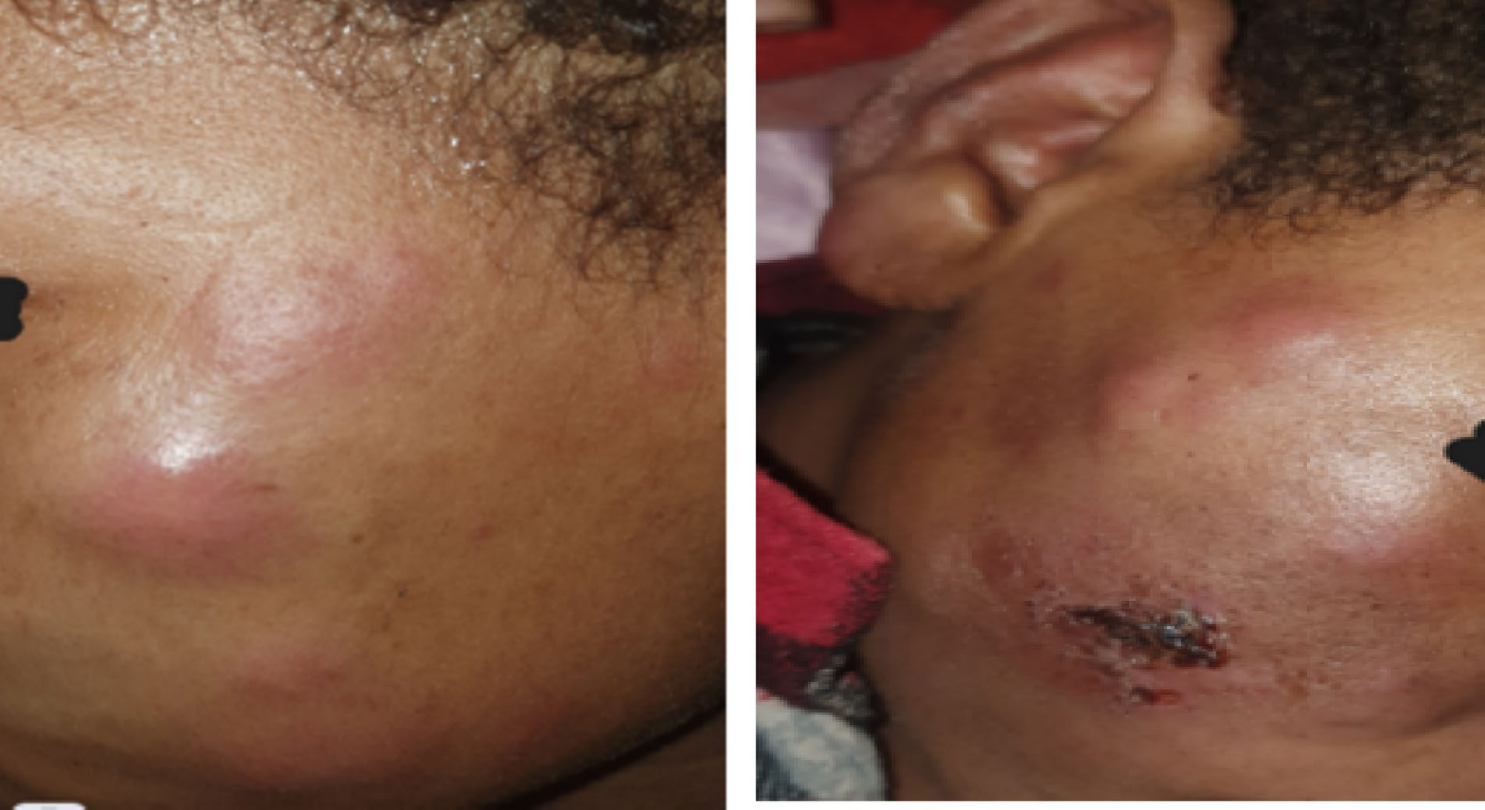
**(A,B)** There are multiple erythematous nodules and plaques scattered over the cheeks bilaterally; there is also eyebrow loss (madarosis) extending from the lateral to medial aspect (the images were taken with consent).

On physical examination, the patient appeared acutely ill and in pain, with a blood pressure of 100/60 mmHg, a pulse rate of 110 beats per minute, a respiratory rate of 22 breaths per minute, and an axillary temperature of 38.9°C. Conjunctival pallor was also noted. Dermatologic examination revealed multiple erythematous nodular lesions with poorly defined borders and infiltrated plaques on the face and hands bilaterally (Figure 1).

**Figure 2:**
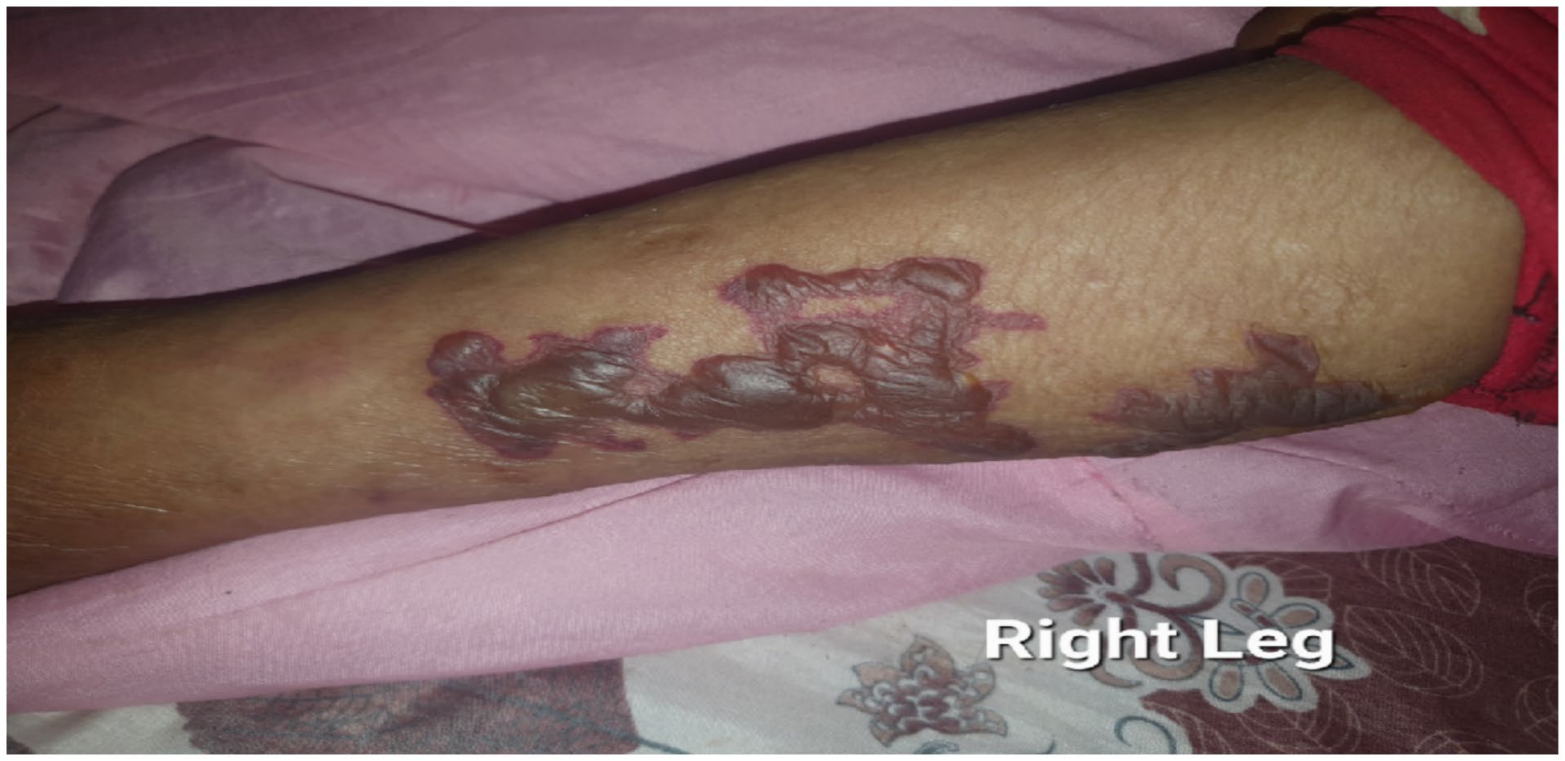
There are hemorrhagic flaccid bullae and vesicles scattered over the medial side of the right leg with the absence of type I Nikolsky sign to emphasize its diagnostic characteristics.

There was also a non-blanching bullous lesion over the right leg with a negative Nikolsky sign (Figure 2). The overall description agrees with the World Health Organization (WHO) definition for leprosy disability grade 1.

**Figure 3:**
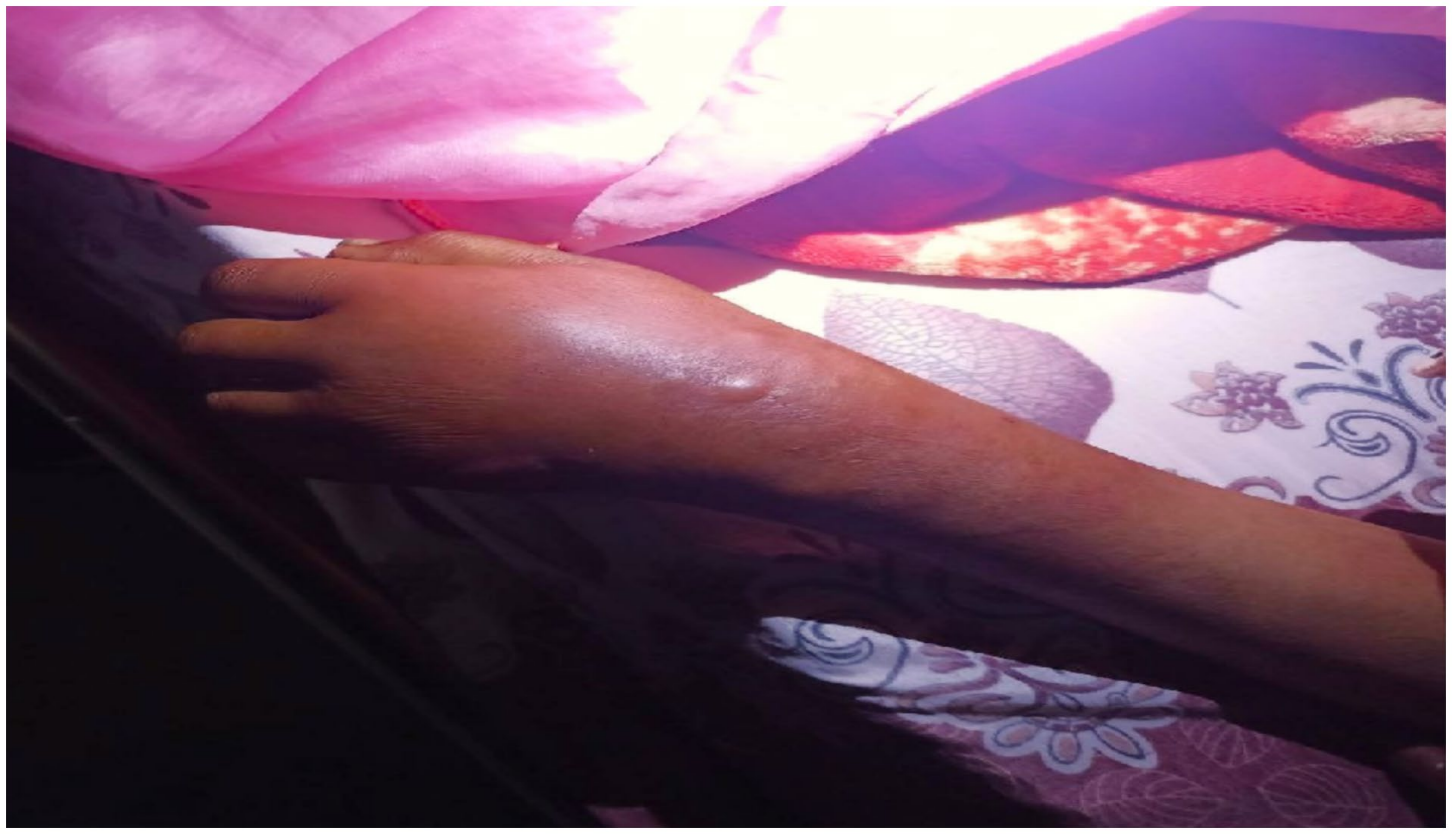
Image shows a left forearm with a prominent, inflamed area. The skin in this region is raised and discolored, with a small blister visible at the center. The surrounding skin appears red and swollen, indicating a possible localized infection or irritation.

The left forearm was swollen and tender to touch, with stretched and shiny overlying skin and a central blister indicating localized inflammation (Figure 3). The ultrasound scan of the hand revealed a minimal superficial hypoechoic collection on the dorsal side, with the deepest pocket measuring 0.4 cm, extending from the radial styloid to the metacarpophalangeal joint of the fifth finger. There was also increased echogenicity and thickness in the subcutaneous tissue, suggestive of an abscess.

**Figure 4:**
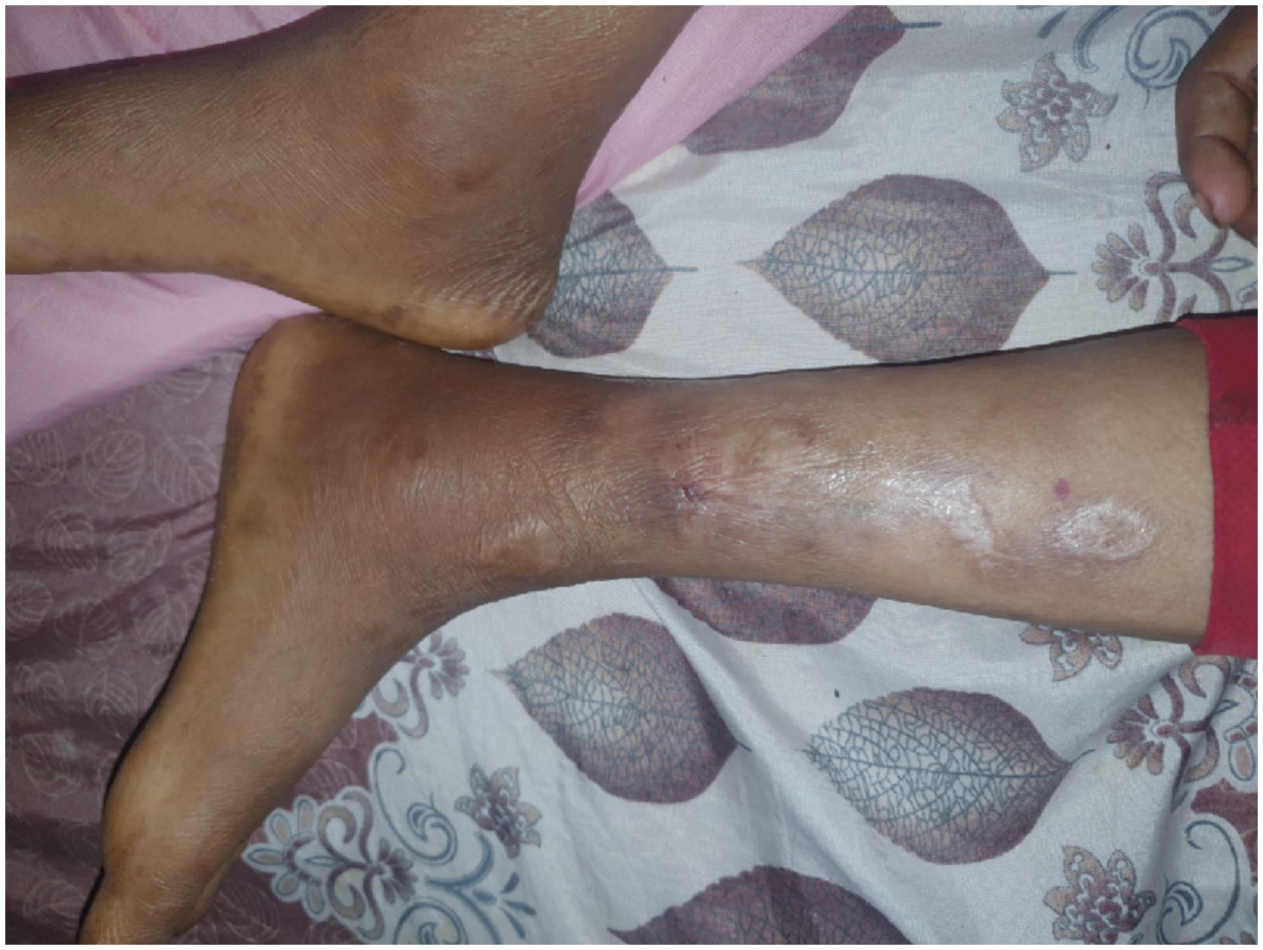
There are multiple hypopigmented and erythematous patches and macules on the medial aspect of the left legs.

There was a hypopigmented skin lesion over the medial aspect of the left leg and thigh bilaterally with decreased sensation over the area (Figure 4).

**Figure 5:**
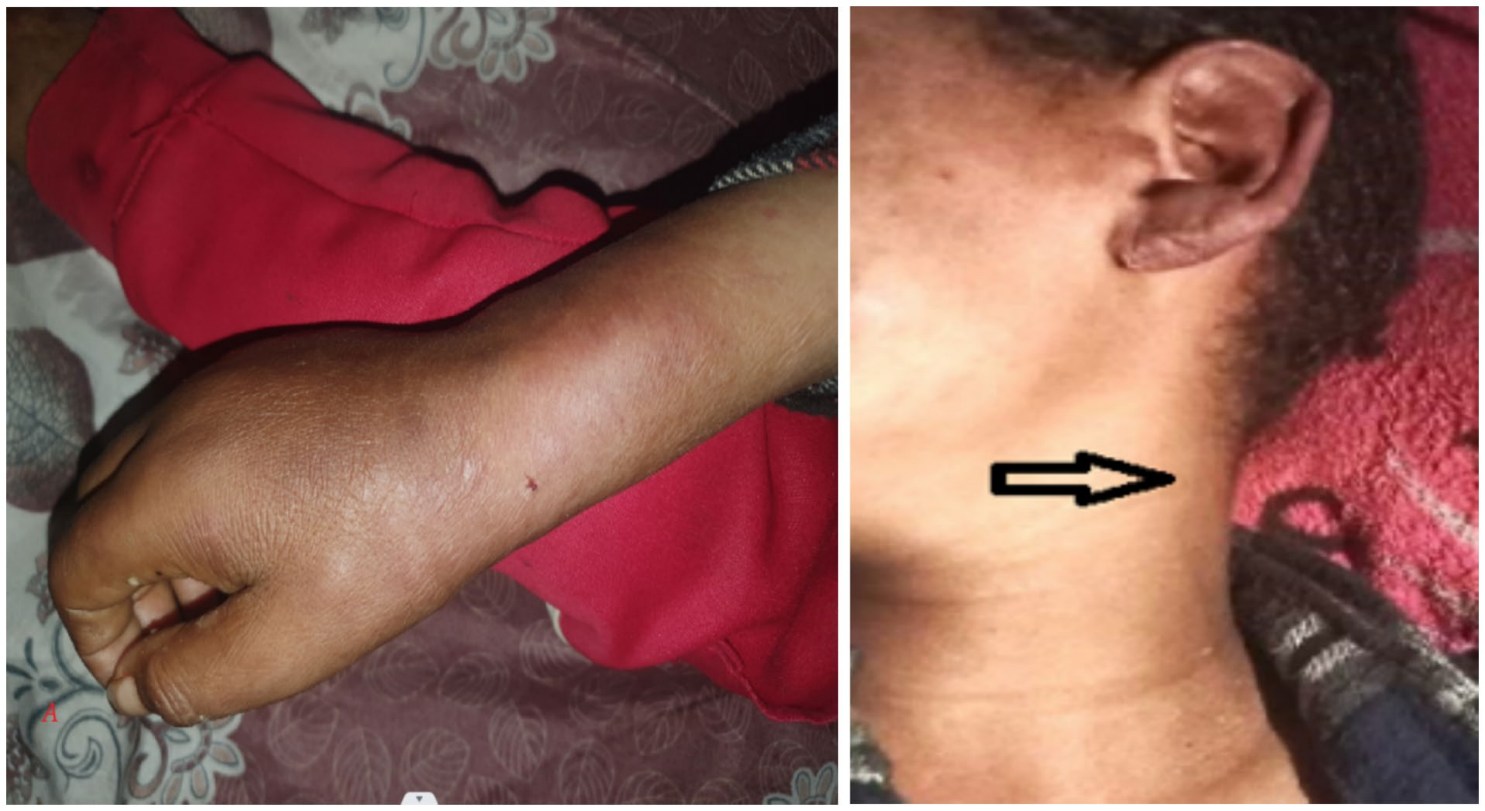
**(A,B)** There are bloody crustations overlying a dirty-based ulcer on the left ear pinna and erythematous nodules on the left lateral side of the face. There is also a visible enlarged left great auricular nerve crossing over the left sternocleidomastoid muscle (the arrowhead), and multiple erythematous nodules scattered over the extensor aspect of the right hand and forearm are noted.

There were bilateral ulnar and radial cutaneous nerve enlargement, saddle nose, madarosis, and tenderness at the olecranon area (Figure 5).

**Figure 6:**
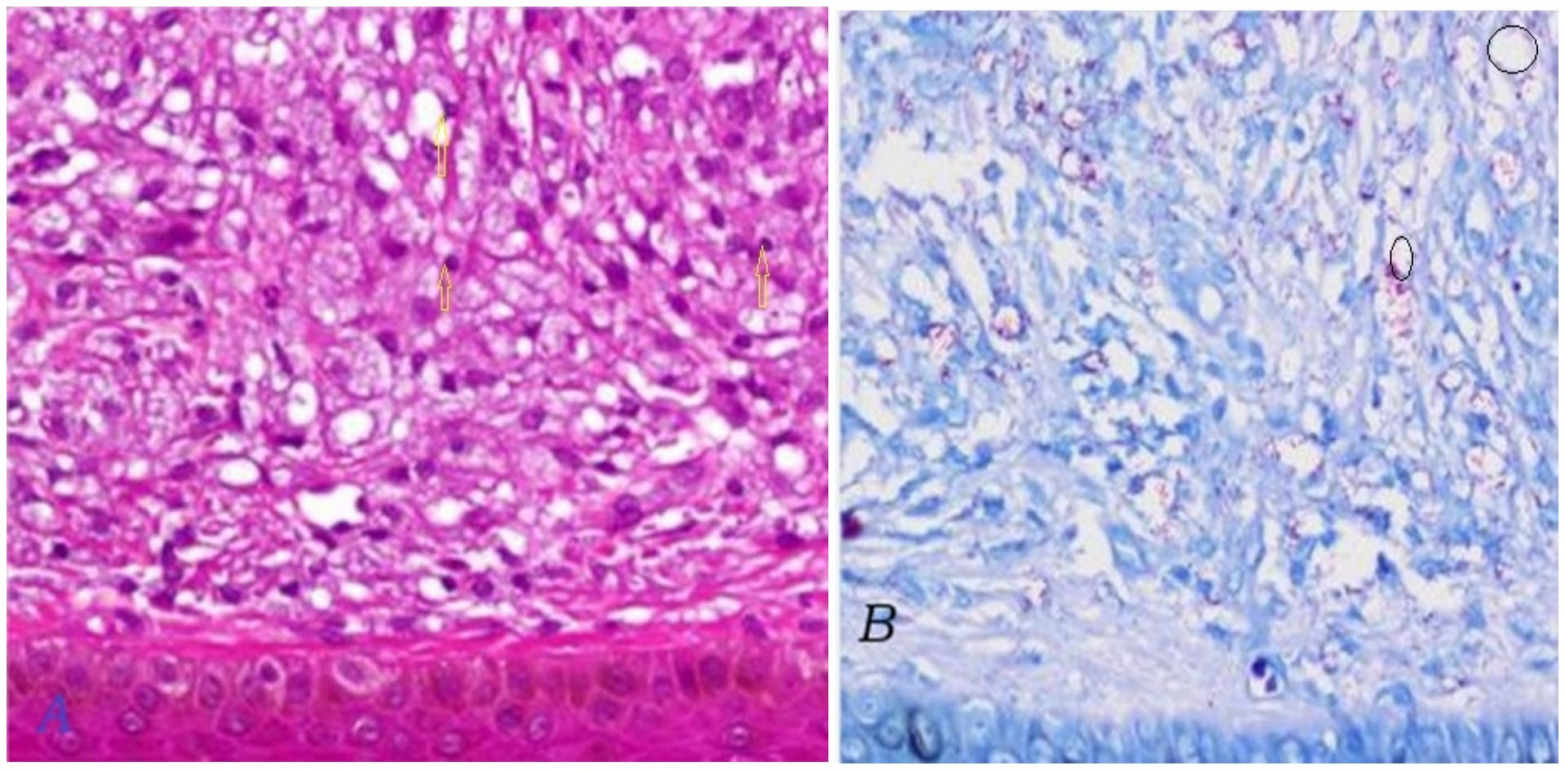
**(A,B)** The figure depicts the histopathological findings from a skin biopsy obtained during the patient’s admission. **(A)** Demonstrates the dermis extensively infiltrated by sheets of foamy histiocytes, characterized by their pale, vacuolated cytoplasm. Notably, well-formed granulomas are absent in this specimen (yellow arrow). **(B)** Highlights the results of an acid-fast stain, revealing a dense population of acid-fast bacilli packed within the foamy macrophages, a hallmark feature indicative of lepromatous leprosy (depicted with circles).

The skin slit smear was taken for acid-fast bacilli, which turned positive for leprosy (Figures 6A,B). Complete blood count and peripheral morphology revealed hypochromic red blood cells with significant anisocytosis and neutrophils with toxic granules, suggestive of microcytic hypochromic anemia with neutrophilic leukocytosis, while other investigations including abdominal ultrasound and chest X-rays were non-revealing.

With the diagnosis of lepromatous leprosy with a secondary reaction, the patient was treated with an initial dose of oral prednisolone at a daily dosage of 40 mg. Cloxacillin was also administered to treat a hand abscess. After controlling the acute symptoms, the patient was started on a combination of oral daily 100 mg of dapsone and 50 mg of daily clofazimine with oral monthly 600 mg of rifampicin to be continued for 12 months, according to the Ethiopian national guideline (8). This multidrug regimen treatment was initiated after 1 week of admission, that is, on his 3rd week of onset of symptoms, and the patient was advised for further follow-up for treatment response.

Follow-up, which is based on the monthly scheduled visit, focuses on educating the patient about the importance of taking medications regularly, the major side effects of the drugs, and the signs and symptoms of possible reactions. The patients should be instructed to report immediately if they encounter/notice any problem/complication while on treatment, and nerve function tests should be performed to detect nerve function damage early to prevent the occurrence of disability (8), as was done for our patient.

Though treatment outcomes are assessed after the patient completes a total of 12 months of MDT within a maximum period of 15 months according to the local guideline (8), response to severe reactions that are better addressed with steroid treatment, as in our case, should be assessed within 4 weeks of treatment initiation, with the patient being advised to return to the tertiary hospital if the reaction worsened and did not improve, or other complications arose. Fortunately, these reactions improved, with no additional worsening recorded in our patient in the 4th week after starting medication. A comprehensive evaluation was conducted on the patient’s siblings and other close family members, but no significant findings suggestive of leprosy were identified. The location and number of family members potentially at risk of transmission were reported to the local leprosy control center to facilitate further screening and follow-up for all close contacts.

## Discussion

Leprosy is a contagious chronic disease caused by *Mycobacterium leprae*, an obligate intracellular bacillus that mostly affects the skin, nerves, and mucous membranes, that spreads from person to person via close contact with those who have a high bacillary index and have not been treated (9, 10). *M. leprae* can be found in skin lesions, breast milk, the environment, and animals, although the respiratory tract is the primary mode of infection (10–13). During disease progression, reactions may develop that, if not treated properly, can cause serious damage to the peripheral nerves, resulting in physical limitations, which are the primary source of the disease’s stigmatization (13).

According to the leprosy case diagnosis, a person is deemed to have leprosy if at least one of the following signs exists: definite loss of sensation in a hypopigmented or reddish skin patch, a thickened or enlarged peripheral nerve with loss of sensation and/or weakness of the muscles supplied by that nerve, or the presence of acid-fast bacilli in a slit-skin smear (12), of which at least three were present in this case report, confirming the diagnosis. The patient’s presentation with a combination of dermatologic, neurologic, and systemic manifestations emphasizes the diverse clinical spectrum of the disease. Notably, the presence of diffuse nodular and bullous lesions, peripheral neuropathy, and systemic inflammatory symptoms such as fever and leukocytosis is consistent with a severe form of multibacillary leprosy, which is likely complicated by a type 2 lepra reaction (erythema nodosum leprosum), though the Lucio phenomenon, which is an uncommon reactional state seen in patients with untreated diffuse lepromatous leprosy, is another possibility. Histopathological features include colonization of endothelial cells by acid-fast bacilli and endothelial proliferation in medium-sized vessels of the mid-dermis. It also involves vasculitis, with or without thrombosis, affecting small vessels in the superficial dermis. This condition typically responds well to anti-leprosy therapy within a few weeks. In severe cases, high-dose steroids may be necessary. However, the presence of localized ulcers, neuritis, and fever in our patient argues against Lucio phenomenon as the diagnosis (10–13).

These reactions can be life-threatening if not promptly managed, as they involve severe inflammation affecting multiple organs, primarily driven by an exaggerated immune response to *Mycobacterium leprae* (6). This case presentation emphasizes the importance of early identification and comprehensive treatment in preventing long-term complications.

The clinical signs of leprosy are used not only to diagnose the disease but also to classify it. These manifestations are essentially the product of the host’s cellular immunological response. Depending on the current clinical classifications, which use the bacilloscopic index, leprosy is categorized into subtypes to facilitate the implementation of multidrug treatment (MDT) in primary care settings (14–17).

The hallmark of lepromatous leprosy (LL) is erythematous-hypopigmented, mildly edematous macules with margins that gradually become hazy compared to normal skin, enlarge, and combine to form large, potentially generalized edematous areas. Papules and nodules (hansenomas or lepromas) may form on edematous areas. Madarosis, xerosis, extremity edema, and palmar and plantar cyanoses have all been observed. These symptoms are often bilateral and symmetrical. Thickening of peripheral nerves with bilateral loss of sensation is also typical (10, 14), and all of these characteristics were present in our case, defining the patient as having lepromatous leprosy. Cutaneous ulcers, which were also present in this case, as well as plantar trophic ulcers with bone loss, develop in LL patients who do not receive appropriate treatment or in those who do not start management at all, as in our case. After summarizing all examinations and investigations, the Lucio phenomenon, which is an uncommon reactional state seen in patients with untreated diffuse lepromatous leprosy, was considered as one of the possibilities. The Lucio phenomenon is caused by *M. lepromatosis*, a closely related mycobacterium that was first identified as a new leprosy-causing agent in 2008 (18). *M. lepromatosis* causes up to 50% of leprosy cases in the western Pacific coastal states of Mexico, but it is extremely rare in other countries and has not been reported in Africa (18). It is characterized by a purpuric macule with many and wide areas of ulceration and bizarre-patterned, angulated borders that primarily affect extremities. Histopathological features include colonization of endothelial cells by acid-fast bacilli, endothelial proliferation of medium-sized vessels of the mid-dermis, and vasculitis with or without thrombosis of the small vessels of the superficial dermis and responds effectively within the few weeks of anti-leprosy therapy and high-dose steroids, which might be required in severe instances; though the localized ulcers, neuritis, and presence of fever in our patient are against the Lucio phenomenon, supporting the most common clinical manifestation of type II reaction, erythema nodosum leprosum (ENL), which occurs mostly in LL patients; however, it is also prevalent among BL patients (8, 19), as in our case. In this case, the presence of systemic symptoms such as fever and joint swelling could have initially pointed to other differential diagnoses such as bacterial sepsis, autoimmune diseases, or systemic infections. However, the characteristic skin lesions, sensory disturbances, and nerve enlargement strongly suggested leprosy. However, previous studies from Ethiopia have highlighted a lack of awareness and expertise among healthcare providers (20). In this patient, the diagnosis was confirmed through a skin slit smear positive for acid-fast bacilli, a gold standard in diagnosing multibacillary leprosy. The positive smear, coupled with the clinical signs of diffuse nodular lesions, sensory impairment, and peripheral nerve thickening, pointed toward a severe, active infection requiring urgent intervention (4, 7, 18). The patient’s hematologic findings, including microcytic hypochromic anemia and neutrophilic leukocytosis, likely indicated systemic inflammation secondary to the leprosy reaction (5, 7).

Leprosy is typically treated as an outpatient using WHO-standardized regimens, which consist of three first-line drugs known as multidrug treatment (MDT): dapsone, rifampicin, and clofazimine, which has an important anti-inflammatory action and is used in type 2 reactions as a steroid-sparing agent (7, 12, 20–22). Expected complications that could arise could be due to the disease itself—leading to permanent damage to bones and nerves—and as a result of drugs used to treat the disease, which include reports of liver toxicity, shock, dyspnea, hemolytic anemia, renal failure, flushing, rash and pruritus, decreased appetite, nausea, vomiting, diarrhea, abdominal pain, malaise, loss of appetite, jaundice, purpura, epistaxis, thrombocytopenia, and psychosis, though these are generally uncommon (23–29).

After stabilization, the patient was advised on adherence, and treatment completion of the multidrug therapy (MDT), according to national guidelines, which is crucial to achieving long-term disease control and preventing relapse. This case underscores the ongoing burden of leprosy and continued community transmission in Ethiopia, particularly among children, as evidenced by this patient’s age. The high prevalence of disability grade II at diagnosis in Ethiopia (4, 7), along with reports of knowledge gaps among healthcare providers (4, 7), indicates a critical need for strengthened leprosy control measures and continued vigilance in identifying and managing leprosy cases.

## Conclusion

This case illustrates the critical role of early recognition and multidisciplinary management in leprosy, particularly in endemic areas. The complexity of the presentation and the associated complications demonstrate the need for ongoing vigilance among healthcare providers. Public health initiatives aimed at improving diagnostic capabilities and expanding leprosy services are vital for reducing the disease burden in countries such as Ethiopia.

## Data Availability

The original contributions presented in the study are included in the article/supplementary material, further inquiries can be directed to the corresponding author.
